# Chemical characterization and antioxidant potential of volatile oil from an edible seaweed *Porphyra tenera* (Kjellman, 1897)

**DOI:** 10.1186/s13065-017-0259-3

**Published:** 2017-04-14

**Authors:** Jayanta Kumar Patra, Se-Weon Lee, Yong-Suk Kwon, Jae Gyu Park, Kwang-Hyun Baek

**Affiliations:** 1 Research Institute of Biotechnology & Medical Converged Science, Dongguk University-Seoul, Ilsandong-gu, Goyang-si, Gyeonggi-do 10326 South Korea; 2 International Technology Cooperation Center, RDA, Jeonju, 54875 Republic of Korea; 3 Department of Biotechnology, Yeungnam University, Gyeongsan, Gyeongbuk 38541 Republic of Korea; 4 Pohang Center for Evaluation of Biomaterials, Pohang Technopark Foundation, Pohang, 37668 Republic of Korea

**Keywords:** Antioxidant, Chemical composition, Volatile oil, *Porphyra tenera*, Seaweed

## Abstract

**Background:**

*Porphyra tenera* (Kjellman, 1897) is the most common eatable red seaweed in Asia. In the present study, *P. tenera* volatile oil (PTVO) was extracted from dried *P. tenera* sheets that were used as food by the microwave hydrodistillation procedure, after which the characterization of its chemical constituents was done by gas chromatography and mass spectroscopy and its antioxidant potential was evaluated by a number of in vitro biochemical assays such as 1,1-diphenyl-2-picrylhydrazyl (DPPH) free radical scavenging, nitric oxide (NO) scavenging, superoxide radical scavenging, 2,2′-azino-bis(3-ethylbenzothiazoline-6-sulphonic acid) (ABTS) radical scavenging, hydroxyl radical scavenging and reducing power assay and inhibition of lipid peroxidation.

**Results:**

A total of 30 volatile compounds comprising about 99.4% of the total volume were identified, of which trans-beta-ionone (20.9%), hexadecanoic acid (9.2%) and 2,6-nonadienal (8.7%) were present in higher quantities. PTVO exhibited strong free radical scavenging activity by DPPH scavenging (44.62%), NO scavenging (28.45%) and superoxide scavenging (54.27%) at 500 µg/mL. Similarly, it displayed strong ABTS radical scavenging (IC_50_ value of 177.83 µg/mL), hydroxyl radical scavenging (IC_50_ value of 109.70 µg/mL), and moderate lipid peroxidation inhibition activity (IC_50_ value of 231.80 µg/mL) and reducing power (IC_0.5_ value of 126.58 µg/mL). PTVO exhibited strong antioxidant potential in a concentration dependent manner and the results were comparable with the BHT and α-tocopherol, taken as the reference standard compounds (positive controls).

**Conclusions:**

Taken together, PTVO with potential bioactive chemical compounds and strong antioxidant activity could be utilized in the cosmetic industries for making antioxidant rich anti-aging and sun-screen lotion and in the food sector industries as food additives and preservatives.

## Background

Reactive oxygen species (ROS) including hydrogen peroxide, hydroxyl radical, superoxide anion, and singlet oxygen are continuously generated in the biological systems during the normal breakdown of oxygen or treatment with exogenous agents [1, 2]. Inappropriate scavenging of these ROS results in oxidative damage to lipids, proteins and DNA. These effects are linked to a number of pathological processes such as atherosclerosis, diabetes, neurological disorders and pulmonary dysfunction [3]. Oxidative degradation of lipids plays an important role in causing atherosclerosis, ageing and carcinogenesis in humans [4–7].

In the food industry, the oxidation of lipids is one of the most important factors that affects and deteriorates the quality of food. There is extensive loss of nutritional values of the raw and processed food products due to the oxidative degradation of lipids. Hence to protect food products from such damages, various synthetic antioxidants such as butylated hydroxylanisol (BHA) and butylated hydroxytoluene (BHT) are generally used [8]. However, the use of synthetic antioxidants has recently been restricted because of their health risks and toxicities [9]. Moreover, synthetic antioxidants such as α-tocopherol and BHT have been reported to be ineffective against the oxidative deterioration in complex food systems such as muscle foods, where both heme proteins and lipoxygenase enzyme are involved in instigation of the oxidation reaction [10]. Similarly, other commercially available natural antioxidants such as ascorbic acid are not effective for the preservation of some foods enriched with long chain omega-3 fatty acids, which are vulnerable to oxidation of lipid [11]. Furthermore, consumer awareness regarding the safety and quality of food has forced the food processing industry to search for alternative sources of antioxidants from natural origins. A number of studies have focused on the use of natural antioxidants from terrestrial plants in food systems to prevent the damage caused by the ROS [12]. Therefore, many plants and their products have been investigated as natural antioxidants and for their potential for use in nontoxic and consumer friendly products.

For centuries, seaweeds belonging to laminariales, chlorophyta and Rhodophyta have been utilized as food supplements and for various medicinal purposes [13]. These seaweeds represent an important economic resource and are consumed as major food products in many Asian countries including Korea, Japan and China [14–18]. The nutrient compositions of seaweeds vary among different species, their habitats of growing, maturity and a number of climatic and environmental conditions [19, 20]. Studies searching for natural products from seaweeds have significantly increased in recent years, and a variety of beneficial compounds with a number of biological activities have been identified in seaweeds [9]. Among antioxidant compounds, astaxanthin, catechins, fucoxanthin, phlorotannins, sulphated polysaccharides and sterols have been isolated from many seaweeds [17, 21–24].

Among various types of seaweed consumed as food, *Porphyra tenera* is the most common and abundantly used in Korea, Japan and China [18]. The genus *Porphyra*, traditionally known as kim in Korea, nori in Japan and zicai in China, is a popular food due to its rich flavor and useful compounds it contains, including vitamins, minerals, protein, and dietary fiber [25–27]. This seaweed also contains various inorganic and organic substances including carotenoids, polyphenols and tocopherols [28]. Although many studies have been conducted to investigate the antioxidant potentials of these seaweeds [17, 18, 29–32]; none have investigated the extraction of volatile oil from *P. tenera* and its usage. In the present study, volatile oil was extracted from the edible seaweed *P. tenera,* its chemical constituents were analyzed and its antioxidant potential were evaluated.

## Results

### Chemical analysis

**Fig. 1:**
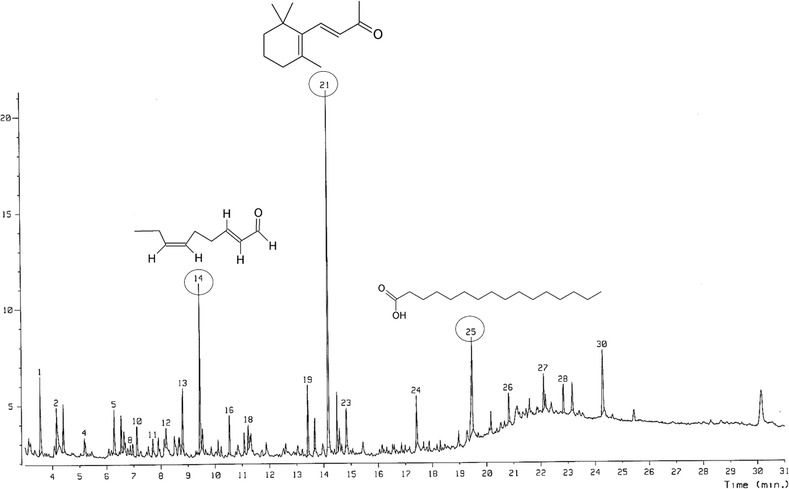
GC–MS spectra of *Porphyra tenera* volatile oil and the chemical structure of three dominant compounds

Volatile oils with a clear yellow color were obtained by the hydrodistillation of a red seaweed, *P. tenera*, with a yield percent of 1.41%. The PTVO obtained were analyzed for their chemical constituents by GC–MS analysis and the results were presented in Table 1 and Fig. 1. A total of 30 volatile compounds comprising 99.4% of the total volume were identified (Table 1). The main compounds identified were fatty acids, ketones, alcohols, aldehydes and monoterpene groups. Among the identified compounds, trans-beta-ionone (20.9%), hexadecanoic acid (9.2%) and 2,6-nonadienal (8.7%) were dominant, accounting for 38.8% of the PTVO.

**Table 1:** GC–MS spectra of *Porphyra tenera* volatile oil (PTVO) with tentative identified compounds

No.	Compounds	SI	RT	RA (%)
1	n-Hexanal	898	3.55	4.7
2	Dimethyl sulfoxide	891	4.15	3.8
3	2-Hexen-1-ol	911	4.40	2.6
4	4-Heptenal	813	5.18	0.7
5	Benzaldehyde	937	6.28	2.8
6	2 Octenal	642	6.53	2.4
7	1-Octen-3-ol	798	6.63	1.2
8	2,4-Heptadienal	697	6.88	0.5
9	n-Octanal	657	6.96	0.6
10	2,4-Heptadienal	811	7.11	2.1
11	Benzene acetaldehyde	844	7.70	0.8
12	E,E-2,4-Octadien-1-ol	689	8.20	1.0
13	2-Heptanone	534	8.81	3.8
14	2,6-Nonadienal	836	9.44	8.7
15	Piperitone oxide	665	9.53	1.4
16	beta-Cyclocitral	794	10.53	2.2
17	3,5-Octadiene	591	11.08	0.9
18	3-Dodecyne	667	11.23	1.6
19	Alpha-ionone	834	13.41	4.0
20	Neryl acetone	644	13.67	1.9
21	Trans-beta-ionone	794	14.17	20.9
22	Phenol	883	14.49	2.9
23	2(4H)-Benzofuranone	864	14.84	2.7
24	Tetradecanoic acid	818	17.42	3.2
25	Hexadecanoic acid	785	19.47	9.16
26	2-Hexadecen-1-ol	728	20.82	1.9
27	Benzoic acid	347	2.11	2.2
28	Hexanoic acid	422	22.85	2.0
29	9-Octadecenamide	501	23.16	1.9
30	Azetidine	449	24.28	5.6

*No.* compound number in order of elution, *SI* library search of purity value of a compound, *RT* retention time (min), *RA* relative area

### Antioxidant potential of PTVO

The antioxidant potential of PTVO was assessed by various in vitro assays, namely DPPH free radical scavenging, NO scavenging, superoxide radical scavenging, ABTS radical scavenging, hydroxyl radical scavenging and reducing power assay in addition to inhibition of lipid peroxidation.

#### DPPH free radical scavenging activity

**Fig. 2:**
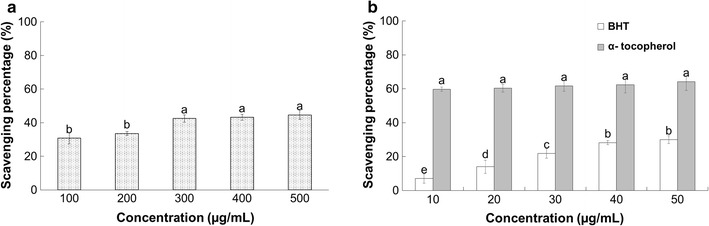
DPPH radical scavenging potential of **a** *Porphyra tenera* volatile oil (PTVO) and **b** BHT and α-tocopherol as the reference compound. *Different superscripts in each column* indicate significant differences in the mean at P < 0.05

The DPPH scavenging potential of PTVO and standard reference compound (positive controls), BHT and α-tocopherol, is presented in Fig. 2. PTVO exhibited 44.62% DPPH free radical scavenging potential at 500 µg/mL, and the reference compounds BHT and α-tocopherol exhibited 30 and 64.15% inhibition at 50 µg/mL, respectively (Fig. 2).

#### Nitric oxide scavenging activity

**Fig. 3:**
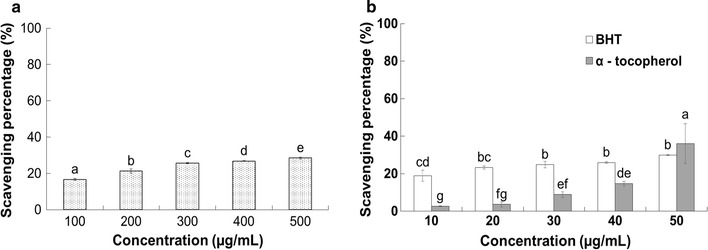
Nitric oxide scavenging potential of **a** *Porphyra tenera* volatile oil (PTVO) and **b** BHT and α-tocopherol as the reference compound. *Different superscripts in each column* indicate significant differences in the mean value at P < 0.05

The nitric oxide scavenging potential of PTVO and BHT and α-tocopherol taken as the positive controls, is presented in Fig. 3. The results indicated that PTVO exhibited a moderate activity of 28.45% scavenging at 500 µg/mL, whereas the reference compounds, BHT and α-tocopherol, exhibited 29.86 and 35.98% scavenging at 50 µg/mL, respectively (Fig. 3).

#### Super oxide anion radical scavenging activity

**Fig. 4:**
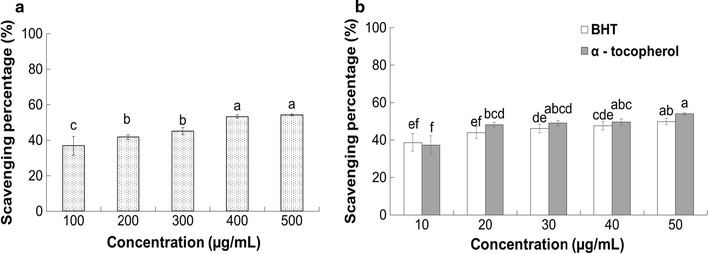
Superoxide radical scavenging potential of **a** *Porphyra tenera* volatile oil (PTVO) and **b** BHT and α-tocopherol as the reference compound. *Different superscripts in each column* indicate significant differences in the mean value at P < 0.05

The superoxide radical scavenging effect of PTVO and BHT and α-tocopherol taken as the positive controls, is presented in Fig. 4. PTVO exhibited a high superoxide radical scavenging activity of 54.27% at 500 µg/mL (Fig. 4), while the reference compounds, BHT and α-tocopherol, exhibited 49.89 and 54.03% scavenging at 50 µg/mL, respectively (Fig. 4).

#### ABTS radical scavenging activity

The ABTS free radical scavenging potential of PTVO and the reference compounds, BHT and α-tocopherol taken as the positive controls, is shown in Table 2. The IC_50_ value of PTVO was 177.83 µg/mL, whereas those of BHT and α-tocopherol were 26.70 and 21.36 µg/mL, respectively. The IC_50_ value of PTVO was higher than those of the reference compounds representing less activity of PTVO.

**Table 2:** Antioxidant activity of *Porphyra tenera* volatile oil (PTVO)

Antioxidant activity	PTVO	BHT	α-Tocopherol
ABTS radical scavenging activity*	177.83 ± 0.85	26.70 ± 0.89	21.36 ± 0.27
Hydroxyl radical scavenging*	109.70 ± 0.19	26.54 ± 0.67	26.45 ± 0.18
Inhibition of lipid peroxidation*	231.80 ± 0.94	47.73 ± 0.50	47.01 ± 0.88
Reducing power**	126.58 ± 0.02	30.19 ± 0.02	25.14 ± 0.04
Phenol content***	4.01 ± 0.66		

* IC_50_ concentration of extract (µg/mL) showing 50% scavenging potential

** IC_0.5_ concentration of extract (µg/mL) showing 0.5 O.D. value at 700 nm

*** Phenol content in mg/g gallic acid equivalent

#### Hydroxyl radical scavenging activity

The hydroxyl radical scavenging potential of PTVO, BHT and α-tocopherol taken as the positive controls are also presented in Table 2. The results showed that PTVO had an IC_50_ value of 109.70 µg/mL, which is represents its high hydroxyl radical scavenging potential. The reference compounds, BHT and α-tocopherol, contained IC_50_ values of 26.54 and 26.45 µg/mL, respectively.

#### Inhibition of lipid peroxidation activity

The inhibitory effect of PTVO, BHT and α-tocopherol taken as the positive controls against lipid peroxidation is summarized in Table 2. PTVO had an IC_50_ value of 231.80 µg/mL, while BHT and α-tocopherol had values of 47.73 and 47.01 µg/mL, respectively.

#### Reducing power activity and total phenol content

The reducing power of PTVO was presented in terms of the IC_0.5_ value in Table 2. PTVO has an IC_0.5_ value of 126.58 µg/mL, while BHT and α-tocopherol taken as the positive controls had values of 30.19 and 25.14 µg/mL, respectively. The total phenol content of PTVO was found to be 4.01 mg/g gallic acid equivalent based on the standard calibration curve of gallic acid taken as reference standard (Table 2).

## Discussion

The volatile compounds identified in PTVO (Table 1) were previously being reported to be medicinally important with anticancer, antioxidant and anti-inflammatory potential [33–36]. 2,6-nonadienal is most commonly used as a flavor and aroma compound by the food industries [33, 37]; and trans-beta-ionone has been reported to possess antiproliferative and antioxidant potential [38]. The presence of these beneficial compounds in the PTVO could make it a potential candidate for application in the food sector, cosmetic and pharmaceutical industries. Similar types of compounds have also been identified in the volatile liquids from different plant and seaweed species [39–43]. Previously, Kajiwara et al. [44], have also reported on the identification of major volatile compounds from the conchocelis-filaments of fresh *P. tenera*. In the present study, the volatile oils were identified from the dry sheets of *P. tenera* commercially available in the local markets for eating purpose and it also showed the presence of similar compounds.

PTVO displayed strong antioxidant potential as evident from the number of in vitro assays (Table 2; Figs. 2,3,4). PTVO, BHT and α-tocopherol which were taken as reference standard compound (positive controls), all showed concentration dependent activity (Fig. 2). Different types of bioactive compounds present in PTVO might have donated an extra electron to neutralize the effects of the DPPH free radical as indicated by the change in color of the reaction medium from dark purple to yellow [45]. Various studies have been conducted to investigate the DPPH radical scavenging potential of volatile oils from different terrestrial plants [46–48]; however, few studies have investigated the DPPH radical scavenging activity of volatile oil from seaweeds [49, 50]. The inhibitory effect of PTVO on the DPPH free radical could also be due to termination of the free radical chain reaction of peroxy radicals that propagates lipid peroxidation process [51].

Nitric oxide is reported to be a very unstable radical that produces highly reactive molecules such as NO_2_, N_2_O_4_ and N_3_O_4_ when reacted with oxygen molecules, leading to various physiological disorders such as fragmentation of DNA, lipid peroxidation and cell damage in the body [52, 53]. The moderate nitric oxide scavenging effect of PTVO (Fig. 3) indicates that it could also be used as an effective antioxidant. Superoxide is a relatively stable radical that is generated in living systems and very harmful to the cellular components under oxidative stress [54, 55]. Serious damage to the DNA, proteins and lipids are caused by ROS such as singlet oxygen and hydroxyl radicals which were generated by the superoxide radicals [56]. The strong superoxide scavenging potential of PTVO (Fig. 4) could make it a potential candidate for used as a natural source of antioxidants in food additives. The moderate ABTS radical scavenging activity exhibited by PTVO (Table 2) might have been due to the existence of a number of functional groups in PTVO or the stereoselectivity of the radicals, which could have affected the capacity to react and quench different radicals in the reaction medium [57]. However, the strong hydroxyl radical scavenging potential of PTVO (Table 2) could be attributed to the presence of chemical compounds such as trans-beta-ionone and benzaldehyde (Table 1), which have previously been described to possess antioxidant and antiproliferative activity [38, 58].

Lipid peroxidation is a recognized mechanism process of cellular injury in both plants and animals [59], and is used as an indicator of oxidative stress in different cells and tissue in the body. The lipid oxidation the most important factors that adversely affects the quality of food [9]. Indeed, oxidative degradation of lipids in raw and the processed food is responsible for loss of nutritional value, and plays an essential role in diseases such as ageing, atherosclerosis, and cancer in humans [9, 60]. The inhibition of lipid peroxidation potential of PTVO (Table 2) could be a positive indication of its application in food processing and preservation. The strong reducing power of PTVO (Table 2) could be attributed to the presence of different types of potential antioxidant rich compounds [61]. Phenolic compounds are very important constituents that act as electron donors in free radical reactions because of their scavenging ability [2, 62]. Many studies have shown that the polyphenols extracted from various seaweeds are associated with antioxidant potential and plays an important role in the stabilization of lipid peroxidation [63]. The high phenol content of PTVO (Table 2) could be indicative of its strong antioxidant potential. Many studies of the antioxidant potential of the seaweed species *P. tenera* have previously been reported previously [17, 18, 29–32]; and the present investigation confirmed the strong antioxidant potential of PTVO.

## Conclusions

In conclusion, PTVO extracted from an edible seaweed, *P. tenera*, possesses various types of chemical compounds including high levels of trans-beta-ionone, hexadecanoic acid and 2,6-nonadienal. PTVO exhibited strong antioxidant properties in terms of ABTS, DPPH free radical, NO, hydroxyl radical scavenging and superoxide scavenging in addition to lipid peroxidation inhibition and reducing power. These properties of PTVO could make it a prospective candidate for application in food processing and preservation, as well as in the cosmetic and pharmaceutical industries.

## Methods

### Extraction of volatile oil from *P. tenera* and chemical analysis

The dry, edible seaweed, *P. tenera* (Kjellman, 1897), was purchased from a local market in Gyeongsan, Republic of Korea. The seaweeds were cultivated and dried in Wando Island and distributed by Wandodasima Company (Wando, Republic of Korea). About 250 g of the dry sheets were broken to small irregular pieces by hand and subjected to the extraction of volatile oil by the microwave-assisted hydro-distillation procedure as described in our previous publication [49]. The extracted *P. tenera* volatile oil (PTVO) was then dried over anhydrous sodium sulfate to remove any tress of water and kept in an air tight glass container at 4 °C until further use.

### Chemical analysis of volatile oil from *P. tenera*

Analysis of chemical constituents of the volatile compounds in PTVO was conducted using a gas chromatography–mass spectroscopy (GC–MS) system (JMS 700 MStation, Jeol Ltd., USA) as described in our previous publication [49]. The machine configuration of the GC–MS system includes an Agilent 6890N GC DB-5 MS fused silica capillary column of 30 m × 0.25 mm i.d. with a film thickness of 0.25 µm. For GC–MS detection, an electron ionization system with ionization energy of 70 eV was used. Helium was applied as the carrier gas at a constant flow rate of 1 mL/min. The temperature of the injector and MS transfer line was set at 280 and 250 °C, respectively. At first, the oven temperature was maintained at 50 °C for 2 min, and then it was increased to 250 °C at a rate of 10 °C/min, where it was held for 10 min. Samples (1 µL of 100 times-diluted samples in methanol) were injected manually in splitless mode through the injector. The relative percentages of the constituents of PTVO were expressed as percentages calculated by normalization of the peak area. Identity of the components of PTVOs was assigned by the comparison of their GC retention times on a DB-5 capillary column and similarity index and mass spectra, which were compared to the mass spectra in the computer using the library searches (Wiley and National Institute of Standards and Technology libraries) having more than 62,000 patterns for the GC–MS system and published literature of spectral data whenever possible [44, 64]. The mass spectrum of the unknown component was compared with the spectrum of the known components stored in the NIST library. The identified compound names were the tentative assignments that were made solely on the grounds of MS similarity indices as obtained by the library search in the Wiley and National Institute of Standards and Technology libraries for the GC–MS system and some published literature of spectral data. The relative amounts (RA) of individual components of the PTVO were expressed as the percentages of the peak area relative to the total peak area. The ACD Chemsketch software (http://www.acdlabs.Com/resources/freeware/chemsketch) was used to drawn the chemical structures of some dominant compounds present in the PTVO.

### Evaluation of antioxidant potentials of PTVO

The antioxidant potential of PTVO was evaluated by a number of in vitro assays, DPPH free radical scavenging, nitric oxide scavenging, superoxide radical scavenging, ABTS radical scavenging, hydroxyl radical scavenging and reducing power assay in addition to inhibition of lipid peroxidation. All specific chemicals used for the antioxidant studies were purchased from Sigma-Aldrich (St. Louis, MO, USA).

#### DPPH free radical scavenging assay

The DPPH free radical scavenging potential of PTVO was evaluated as per standard procedure [56]; with slight modification. Briefly, the reaction mixture solution consisted of 50 µL of 0.1 mM DPPH in methanol and 50 µL of different concentrations of PTVO (100–500 µg/mL) that was mixed thoroughly and incubated for 30 min with continuous shaking at 150 rpm at 37 °C in darkness. 50 µL of methanol mixed with 50 µL of 0.1 mM DPPH was taken as the control, and 50 µL of BHT or α-tocopherol at 10–50 µg/mL was taken as the reference standard compound (positive controls). The results were recorded as the scavenging percentage activity calculated by Eq. (1) after measuring the absorbance at 517 nm using a microplate reader (Infinite 200 PRO, Tecan, Mannedorf, Switzerland).

1 $${\text{Scavenging percentage}} \,(\%) = \frac{{Abs_{(control)} - Abs_{(treatment)} }}{{Abs_{(control)} }}\times100$$

where, *Abs*_*(control)*_ or *Abs*_*(treatment)*_ is the absorbance of the control and the treatment, respectively.

#### NO scavenging activity of PTVO

The NO scavenging potential of PTVO was evaluated as per standard procedure [65]. Briefly, 100 µL of different concentrations of PTVO (100–500 µg/mL) or BHT or α-tocopherol (10–50 µg/mL) taken as reference standard compound (positive controls) were mixed with 100 µL of 10 mM sodium nitroprusside in phosphate buffer saline (pH 7.4), then incubated at 37 °C for 60 min in light. After incubation, 75 µL aliquots of the reaction mixture solution in separate vials were added with 75 µL of Griess reagent (1.0% sulfanilamide and 0.1% naphthyl ethylene diamine dihydrochloride), mixed vigorously and incubated for 30 min in the dark at 25 °C. The absorbance of the reaction mixture solution was then measured at 546 nm using the micro plate reader and the NO scavenging activity was calculated as per Eq. 1.

#### Superoxide radical scavenging activity of PTVO

The superoxide anion scavenging potential of PTVO was evaluated as previously described [66] Briefly, a total of 100 µL of the reaction mixture solution consisted of 40 µL of 0.02 M phosphate buffer (pH 7.4), 10 µL of 15 µM phenazine methosulfate (PMS), 10 µL of 50 µM nitroblue tetrazolium (NBT), 10 µL of 73 µM nicotinamide adenine dinucleotide (NADH), and 30 µL of PTVO (100–500 µg/mL) or BHT/α-tocopherol (10–50 µg/mL) taken as reference standard compound (positive controls). The reaction mixture solution containing 30 µL of methanol was used as the control. The reaction mixture solution was mixed meticulously and incubated for 1 h at room temperature in the dark, after which the levels were calculated from the absorbance at 560 nm using Eq. 1.

#### ABTS radical scavenging activity of PTVO

The ABTS radical scavenging potential of PTVO was evaluated by a previously described standard procedure [67]. Prior to use, the ABTS stock solution was prepared by mixing 2.6 mM potassium persulfate and 7.4 mM ABTS at a ratio of 1:1, then incubated for 12 h in darkness. A total of 150 µL of the reaction mixture solution contained 135 µL of ABTS stock solution and 15 µL of different concentrations of PTVO (100–500 µg/mL) or BHT/α-tocopherol (10–50 µg/mL) taken as reference standard compound (positive controls). The reaction mixture solution was mixed appropriately and incubated for 2 h in dark at room temperature. Reaction mixture solution amended with 15 µL of methanol was taken as the control. The absorbance of the reaction mixture solution was measured at 734 nm and the result was calculated in terms of its IC_50_ values (concentration of PTVO required to scavenge 50% of the ABTS radicals) by regression analysis.

#### Hydroxyl radical scavenging activity of PTVO

The hydroxyl radical scavenging potential of PTVO was evaluated as per standard procedure [68]. Briefly, a total of 240 µL of the reaction mixture solution contains 40 µL of 3 mM 2-deoxyribose, 40 µL of 0.1 mM ethylenediamine-tetra acetic acid, 40 µL of 0.1 mM ferric chloride, 40 µL of 2 mM hydrogen peroxide, 40 µL of 0.1 mM ascorbic acid prepared in 20 mM potassium phosphate buffer (pH 7.4) and 40 µL of various concentrations of PTVO (100–500 µg/mL) or BHT/α-tocopherol (10–50 µg/mL) taken as reference standard compound (positive controls). The reaction mixture solution was mixed thoroughly and incubated at 37 °C for 45 min, after which 40 µL of 2.8% trichloroacetic acid and 40 µL of 0.5% thiobarbituric acid in 0.025 M sodium hydroxide solution were added and the solution was further incubated for another 15 min at 90 °C. After completion of the reaction, the mixture solution was completely cooled and the absorbance was measured at 530 nm. The results were calculated as IC_50_ values (concentration of PTVO required to scavenge 50% of hydroxyl radicals) based on regression analysis. Reaction mixture solution amended with 40 µL of methanol was taken as control for the experiment.

#### Inhibition of lipid peroxidation

Inhibition of the lipid peroxidation effect of PTVO was determined as per standard procedure [69]. Briefly, a total of 100 µL of the reaction mixture solution contained of 10 µL of 1 mM ascorbic acid in 20 mM phosphate buffer, 10 µL of 1 mM FeCl_3_, 30 µL of PTVO (100–500 µg/mL) or BHT/α-tocopherol (10–50 µg/mL) taken as reference standard compound (positive controls) and 50 µL of bovine brain phospholipids (5 mg/mL). The reaction mixture solution was mixed meticulously and incubated at 37 °C for 60 min. Next, 100 µL of 30% TCA acid, 100 µL of 1% TBA, and 10 µL of 4% BHT were added to it and boiled in a boiling water bath for 20 min. After the reaction was complete, the sample was cooled to room temperature and the absorbance was recorded using a microplate reader at 532 nm. The results are presented as the IC_50_ values calculated by regression analysis. Reaction mixture solution containing 30 µL of methanol was taken as the control mixture for the experiment.

#### Reducing power assay

The reducing power of PTVO was determined using the standard method [70]. Briefly, a total of 150 µL of the reaction mixture solution contained of 50 µL of 1% potassium ferricyanide, 50 µL of 0.2 M phosphate buffer (pH 6.6) and 50 µL of LJEO (100–500 µg/mL) or BHT/α-tocopherol (10–50 µg/mL) taken as reference standard compound (positive controls). The mixture solution was mixed thoroughly and incubated at 50 °C in dark for 20 min, followed by termination of the reaction by the addition of 50 µL of 10% TCA. The total solution was centrifuged at 3000 rpm for 10 min, after which 50 µL of the supernatant was placed in another vial and mixed with 50 µL of distilled water and 10 µL of 0.1% FeCl_3_ solution, and further incubated for another 10 min at room temperature. The absorbance of the solution was measured at 700 nm. The results were represented as the IC_0.5_ values (concentration of PTVO required to obtain a 0.5 O.D. value) calculated by regression analysis.

#### Total phenolic content

The total phenolic content in PTVO was determined according to the Folin-Ciocalteu’s phenol method [56]. The reaction mixture solution had a total volume of 100 µL, consisting of 50 µL PTVO (0.1 mg/mL) and 50 µL 50% Folin-Ciocalteu reagent. The mixture solution was mixed thoroughly and incubated for 5 min at 25 °C in dark. Next, 100 µL of 20% Na_2_CO_3_ solution was added to the reaction mixture solution slowly and further incubated for 20 min at 25 °C in dark. The absorbance of the solution was measured at 730 nm and the phenolic content of PTVO was calculated on the basis of standard calibration curve generated from gallic acid (5–50 µg/mL), which was taken as the reference compound.

### Statistical analysis

Statistical analysis of the results was accompanied by one-way analysis of variance (ANOVA) followed by Duncan’s test at *P* < 0.05 using the Statistical Analysis Software (SAS) (Version: SAS 9.4, SAS Institute Inc., Cary, NC).
